# Maximization of Power Density of Direct Methanol Fuel Cell for Greener Energy Generation Using Beetle Antennae Search Algorithm and Fuzzy Modeling

**DOI:** 10.3390/biomimetics8070557

**Published:** 2023-11-20

**Authors:** Ahmed Al Shouny, Hegazy Rezk, Enas Taha Sayed, Mohammad Ali Abdelkareem, Usama Hamed Issa, Yehia Miky, Abdul Ghani Olabi

**Affiliations:** 1 Department of Geomatics, Faculty of Architecture and Planning, King Abdulaziz University, Jeddah 21589, Saudi Arabia; ametaowa@kau.edu.sa (A.A.S.); yhhassan@kau.edu.sa (Y.M.); 2 Department of Electrical Engineering, College of Engineering in Wadi Alddawasir, Prince Sattam bin Abdulaziz University, Al-Kharj 11942, Saudi Arabia; hr.hussien@psau.edu.sa; 3 Department of Chemical Engineering, Faculty of Engineering, Minia University, Minya 61111, Egypt; e.kasem@mu.edu.eg; 4 Department of Sustainable and Renewable Energy Engineering, University of Sharjah, Sharjah P.O. Box 27272, United Arab Emirates; aolabi@sharjah.ac.ae; 5 Fuel Cell Institute, Universiti Kebangsaan Malaysia, Bangi 43600, Malaysia; 6 Department of Civil Engineering, Faculty of Engineering, Minia University, Minya 61519, Egypt; usama.issa@mu.edu.eg; 7 Department of Civil Engineering, College of Engineering, Taif University, Taif 21944, Saudi Arabia

**Keywords:** direct methanol fuel cell, beetle antennae search algorithm, fuzzy modeling, optimization

## Abstract

Direct methanol fuel cells (DMFCs) are promising form of energy conversion technology that have the potential to take the role of lithium-ion batteries in portable electronics and electric cars. To increase the efficiency of DMFCs, many operating conditions ought to be optimized. Developing a reliable fuzzy model to simulate DMFCs is a major objective. To increase the power output of a DMFC, three process variables are considered: temperature, methanol concentration, and oxygen flow rate. First, a fuzzy model of the DMFC was developed using experimental data. The best operational circumstances to increase power density were then determined using the beetle antennae search (BAS) method. The RMSE values for the fuzzy DMFC model are 0.1982 and 1.5460 for the training and testing data. For training and testing, the coefficient of determination (R^2^) values were 0.9977 and 0.89, respectively. Thanks to fuzzy logic, the RMSE was reduced by 88% compared to ANOVA. It decreased from 7.29 (using ANOVA) to 0.8628 (using fuzzy). The fuzzy model’s low RMSE and high R^2^ values show that the modeling phase was successful. In comparison with the measured data and RSM, the combination of fuzzy modeling and the BAS algorithm increased the power density of the DMFC by 8.88% and 7.5%, respectively, and 75 °C, 1.2 M, and 400 mL/min were the ideal values for temperature, methanol concentration, and oxygen flow rate, respectively.

## 1. Introduction 

Global warming, which is mainly caused by the overuse of fossil fuels, is a serious environmental concern with far-reaching repercussions. The extensive use of fossil fuels such as coal, oil, and natural gas emit substantial volumes of greenhouse gases into the atmosphere, mainly carbon dioxide [1,2]. These gases trap heat and cause global temperatures to rise, culminating in the phenomena known as global warming. This, in turn, exacerbates catastrophic weather occurrences and upsets delicate ecological balances [3,4]. Aside from these climatic effects, the health of both individuals and ecosystems is jeopardized. As temperatures rise, heat-related ailments and respiratory diseases become more common. The spread of infectious illnesses can potentially be worsened if climate change affects vectors and ecosystems. It is clear that addressing the core cause of global warming by shifting away from the use of fossil fuels is critical for mitigating its cascading effects on our ecosystem, weather patterns, and overall well-being. There are numerous techniques for combating and managing global warming. These tactics include using renewable energies, improving the efficiency of present energy conversion technology, and pushing forward the development of ecologically acceptable energy conversion systems. The fuel cell is one example of an efficient energy conversion device. Fuel cells have small or insignificant environmental footprints, operate quietly, are compact, and have capabilities ranging from a few watts to large scales. Because of their extraordinary efficiency and versatility, fuel cells are set to play a significant role in the renewable energy sector. Fuel cells have emerged as a cornerstone technology in the expanding global emphasis on sustainable energy solutions, notably the interest in green hydrogen. They are perfect for green hydrogen use due to their high efficiency in turning chemical energy into power with low or no environmental impact. A closed-loop, clean energy cycle is achieved by using renewable energy sources to make hydrogen using electrolysis. The hydrogen converted back into electrical power using furl cell. Therefore, fuel cells are becoming essential components of energy storage systems. The ability of these renewable resources to store surplus energy and release it when needed assures a stable and predictable power supply, bridging the intermittent nature of these renewable resources. Fuel cells are poised to shine as a cornerstone technology, propelling the renewable energy sector forward as the world accelerates its move toward cleaner energy choices.

Low-temperature fuel cells like PEMFCs “proton exchange membrane fuel cells” operate optimally at lower temperatures (typically around 80 °C) when membrane was well hydrated [5,6]. While hydrogen remains the optimum fuel for PEMFCs, concerns about its safety, purity, storage, and transportation have arisen [7,8]. To remove these issues, indirect FCs based on hydrocarbons such as methane (natural gas), methanol, and ethanol have become more common [9,10]. Although these hydrocarbons provide safer and more manageable transportation and storage alternatives than hydrogen, indirect systems are larger and less efficient than PEMFCs. As a result, researchers have focused on direct alcoholic fuel cells as possible alternatives to indirect fuel cell systems [11,12]. At the anode side, direct alcoholic fuel cells use simple alcohols such as methanol [13,14], ethanol [15,16], or propanol [17,18] as fuel. Methanol is the simplest alcohol that, when compared to other alcohols, can be easily electrochemically oxidized at the anode side. Methanol also has a high energy density and can be produced using renewable energy sources. Direct methanol fuel cells have the potential to replace Li ion batteries in portable electronics as well as other secondary batteries in transportation. Methanol crossover and slow methanol oxidation at the anode are two major obstacles to the commercialization of direct methanol fuel cells [19,20]. To manage the methanol crossover, many tactics have been used, including reducing the methanol concentration, utilizing thicker membranes, altering the current Nafion membranes with different fillers, replacing Nafion membranes with others with lower crossover, or using a non-precious cathode catalyst that has high oxygen reduction activity and low or no methanol oxidation activity [21,22]. On the other hand, various mathematical modeling studies have been carried out to analyze all of the parameters that affect cell performance, including methanol crossover. Such models are helpful for fully understating all of the parameters affecting/related to methanol crossover, and thus, they are helpful in deciding the best operating condition at minimum crossover and the highest possible power output [23,24,25].

Massive experimental efforts are being undertaken to address methanol crossover and the sluggish methanol oxidation at the anode and optimize the operating conditions (i.e., methanol concentration, cell temperature, etc.). Deciding the optimal operating conditions experimentally requires massive amounts of effort, time, and costs; therefore, modeling and simulation are considered the best choices to realize this aim [26,27]. Although mathematical and physical modeling succeed to a large extent in modeling various processes, they are usually based on parameters that are sometimes based on assumptions, which could affect the model’s accuracy. Machine learning is an artificial intelligence approach that focuses on creating algorithms [28]. Machine learning techniques are used in engineering applications to evaluate complicated data patterns, optimize processes, and improve decision making across multiple domains. Engineers use machine learning to create more efficient systems, detect equipment breakdowns, streamline industrial processes, and improve overall performance. Neural networks are types of algorithms that are based on the structure and operation of the human brain. They are made up of interconnected nodes, or “neurons,” that are structured in layers. Through a process known as training, neural networks may learn patterns and relationships from data [29,30]. Neural networks are utilized in engineering for tasks such as image and speech recognition, control systems, optimization, and prediction. Convolutional Neural Networks (CNNs) thrive at image analysis, whereas Recurrent Neural Networks (RNNs) excel at sequential data processing, making them useful for tasks such as time series analysis and natural language processing [31,32].

Fuzzy logic is a mathematical framework for dealing with decision making uncertainty and imprecision. Unlike standard binary logic, which categorizes everything as either true or false, fuzzy logic can reflect degrees of truth. In engineering applications, fuzzy logic models and controls systems with ambiguity and vagueness [33,34]. Temperature management in HVAC systems, automatic transmission in vehicles, and expert systems for decision support in many engineering fields are examples of fuzzy logic’s many uses. The authors of the present study have already used fuzzy modeling and optimization to model and optimize various engineering processes, such as bio-methanol production [35], microbial fuel cell operation [36,37], increasing carbon capture capacity [38,39], deciding the optimal operating conditions of PEMFC [40,41], biohydrogen production [42], etc. 

Few works have been discussed modeling and optimizing DMFCs. Wang et al. [43] successfully used ANFIS (“adaptive-network-based fuzzy inference systems”) to model a DMFC stack’s performance using current, methanol concentration, and temperature as input parameters and the output is cell voltage. The accuracy of ANFIS was better than those obtained by ANN. Similarly, using ANFIS and ANN, Hasiloglu et al. [44] modeled a DMFC using temperature, methanol solution flow rate and concentration at the anode, airflow at the cathode, and current as the input parameters, while cell voltage was the output. Using fuzzy logic-based control, Cao et al. [45] optimized the energy consumption in a hybrid energy system composed of a battery and a DMFC, aiming to maximize the efficiency of the DMFC while keeping a high state of charge in the battery. Still, a lot of work needs to be carried out for the proper modeling and optimization of DMFCs. The main goal of the current work was to create a trustworthy fuzzy model to simulate DMFCs. Three process variables were considered to boost the power density of the DMFC: temperature, methanol concentration, and oxygen flow rate. Using experimental data, a fuzzy model of the DMFC was first created. The beetle antennae search (BAS) technique was then used to discover the optimal operational conditions to maximize power density.

The main focus of the present work is:

- Constructing a new fuzzy model for simulating direct methanol fuel cell.
- Applying BAS algorithm for determining the best values of temperature, methanol concentration, and oxygen flow rate.
- Boosting the power density of the DMFC.

## 2. Dataset

The dataset points used for the current study are presented in Table 1. These dataset points present the relationships between the output (maximum Power density (mW/cm^2^)) and three input parameters (temperature, methanol concentration, and oxygen flow rate).

## 3. Methodology

The suggested approach comprises two main stages: modeling and optimization. First, using the experimental data, an accurate model that simulates the DMFC was created via fuzzy logic. The datasets were divided into 70% (training) and 30% (testing). The model precision for tracking the data was confirmed first. Then, the BAS algorithm was used to determine the optimal parameters which improve the power density of the DMFC. Three crucial elements determining the reliability of the model predictions were carefully selected in order to build a robust model and handle the overfitting problem: (1) the modelling tool; (2) the ratio between training and testing data; and (3) the number of training epochs. Because fuzzy logic can handle complex and non-linear datasets, it was chosen as the modelling tool.

### 3.1. Fuzzy Modeling

Fuzzy Inference Systems (FISs) could serve as instruments for approximating intricate system behaviors characterized by vague and non-linear functions. These instruments have the ability to attribute qualitative facets of human understanding to approximate the functions. Typically, Fuzzy Inference Systems (FISs) encompass the following five functional elements [47]:

- An assemblage of fuzzy IF–THEN rules within the rule base. The number of rules can be determined by multiplying the number of inputs. The rules are provided as follows:
  ○ If *α* is *X*_1_ and *β* is *Y*_1_*,* then *γ* is *Z*_1_
  ○ If *α* is *X*_2_ and *β* is *Y*_2_*,* then *γ* is *Z*_2_
  ○ If *α* is *X_n_* and *β* is *Y_n_,* then *γ* is *Z_n_*

Here *α* and *β* are inputs; *γ* is an output; and *X*, *Y*, and *Z* are fuzzy sets.

- A database that outlines the membership functions of each set.
- The decision making method.
- A fuzzification stage that converts the inputs to linguistic variables.
- A defuzzification stage that converts the linguistic variables to outputs.

**Figure 1:**
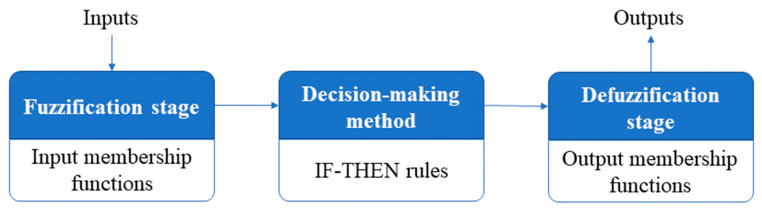
The FIS architecture.

The architecture of the FIS is illustrated in Figure 1.

### 3.2. Beetle Antennae Search Algorithm

The BAS algorithm is based on the natural search behavior of longhorn beetles. This metaheuristic algorithm imitates insect antennae and their unpredictable travel patterns. The beetle uses its two antennae to haphazardly explore surrounding surroundings. In the event that only one antenna detects the target’s odor (where the odor concentration mirrors the objective function, denoted as *f*), the beetle adjusts its course toward that direction. If neither antenna detects the odor, the beetle changes its path to the opposite side. This algorithm efficiently integrates these two elements to navigate and guide its search [48]. 

The new location can be computed as a function of the directional factor d and the location turning of the right and left sides (*x_r_* and *x*_l_), given that *x^t^* represents the beetle’s location at iteration *t*. The following formula can be used to determine *d* in terms of the search space dimensions (*dim*):

(1) $$d=\frac{rand(dim)}{\left|rand(dim)\right|}$$

*x_r_* and *x_l_* can be determined:

(2) $$x_{l}=x^{t}+a^{t}\cdot d$$

(3) $$x_{r}=x^{t}-a^{t}\cdot d$$

*a^t^* is the length of the antennae sensing at *t* and expressed as follows:

(4) $$a^{t}=0.95\cdot a^{t-1}+0.01$$

The new position of the beetle can be determined as follows:

(5) $$x^{t}=x^{t-1}+b^{t}\cdot d\cdot sgn\left(f\left(x_{r}\right)-f(x_{l})\right)$$

*b^t^* (the step size) can be determined as follows:

(6) $$b^{t}=0.95\cdot b^{t-1}$$

## 4. Results and Discussion

To build a concrete model and to overcome the problem of overfitting, fuzzy logic is used. 30 points were used to build the model (21 for training and the rest for testing). The system’s rules, which were developed using the subtractive Clustering Algorithm (SC), were in this work 10. The model was trained until lower RMSE values were obtained. The statistical metrics of the model is shown in Table 2.

**Figure 2:**
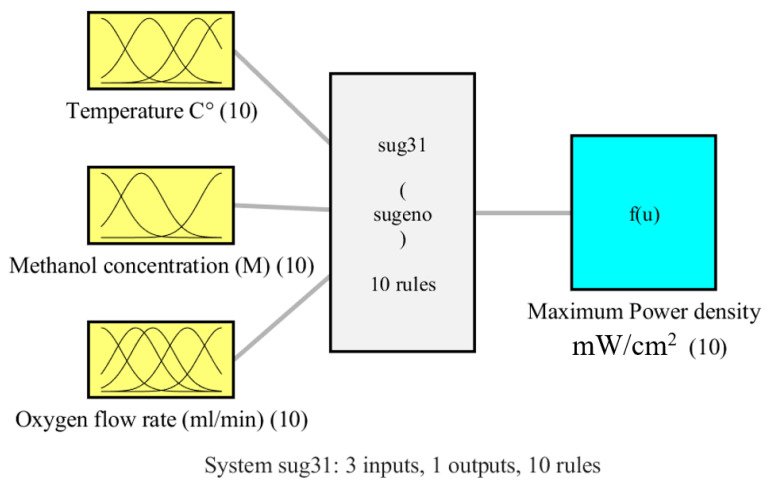
Configuration of DMFC fuzzy model.

**Figure 3:**
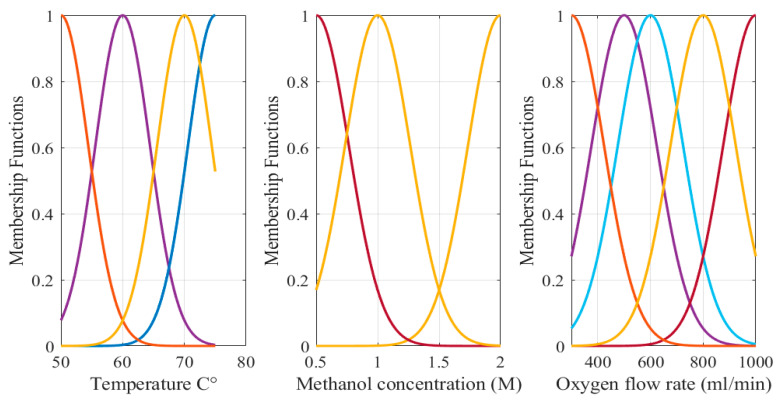
Inputs’ MFs of fuzzy model of DMFC.

As indicated in Table 2, the RMSE values for DMFC model are 0.1982 and 1.5460 for the training and testing data, respectively. The R^2^ values for training and testing are 0.9977 and 0.89. Using fuzzy logic, a 88% reduction in RMSE was achieved compared to ANOVA [46]. Where the RMSE decreased from 7.29 in case of ANOVA to 0.8628 (using fuzzy). The fuzzy model’s low RMSE and high R^2^ values show robustness of the modeling phase. The three-input, single-output fuzzy model of the DMFC is shown in Figure 2, and Figure 3 expresses the Gaussian-shape membership functions.

**Figure 4:**
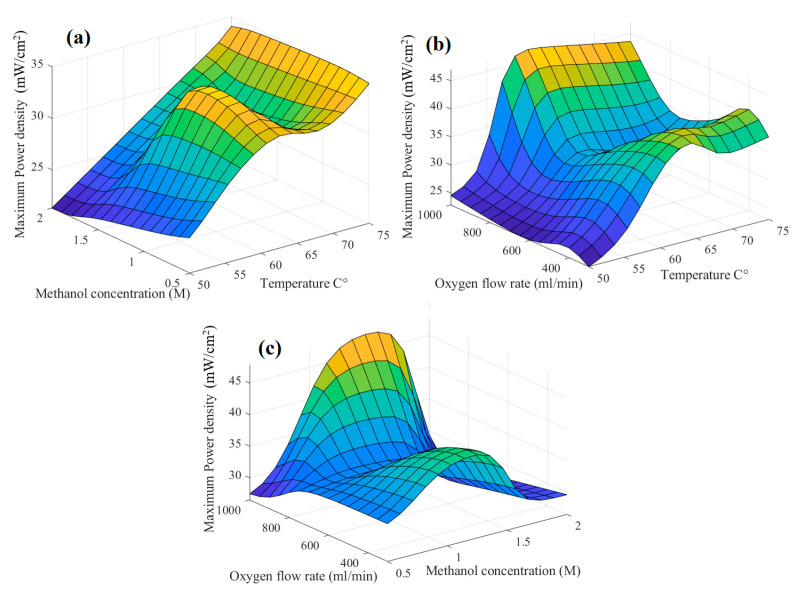
Three-dimensional surface of fuzzy model of power output. (**a**) temperature and methanol concentration; (**b**) oxygen flow rate and temperature; (**c**) oxygen flow rate and methanol concentration.

Figure 4 presents the surface of the DMFC’s fuzzy model when the system’s input–output function is considered for each pair of inputs. Yellow receives the output’s highest value, while dark blue receives the lowest value. The performance of the DMFC at various methanol concentrations (between 0.5 and 1 M) is positively influenced by temperature within the measured range, which is between 50 and 75 °C (Figure 4a). When cell temperature was raised, the electrochemical oxidation and reduction activities at both electrodes, i.e., anode and cathode, is improved, and thus performance [49]. Up to a definite temperature, which is 65 °C in this study, the cell temperature had a favorable impact on the power output of the DMFC when varying oxygen flow rates were applied to the cell’s cathode. However, when the cell temperature increased further, performance degraded (Figure 4b). The influence of cell temperature on the drying of the cathode side with an increase in the cell temperature, especially at higher oxygen flow rates (800 and 1000 mL/min), would result in a slight decrease in power. Figure 4c demonstrates that the cell’s performance improved as the methanol concentration was increased from 0.5 M to 1.5 M. Conversely, the performance declined at higher methanol concentrations, and this was observed at different oxygen flow rates. The effect of the methanol concentration at the various oxygen flow rates is the same as increasing the cell temperature [50] because it is well known that an increase in the methanol concentration would increase the methanol crossover and, consequently, the cell temperature [50] (Figure 4b).

**Figure 5:**
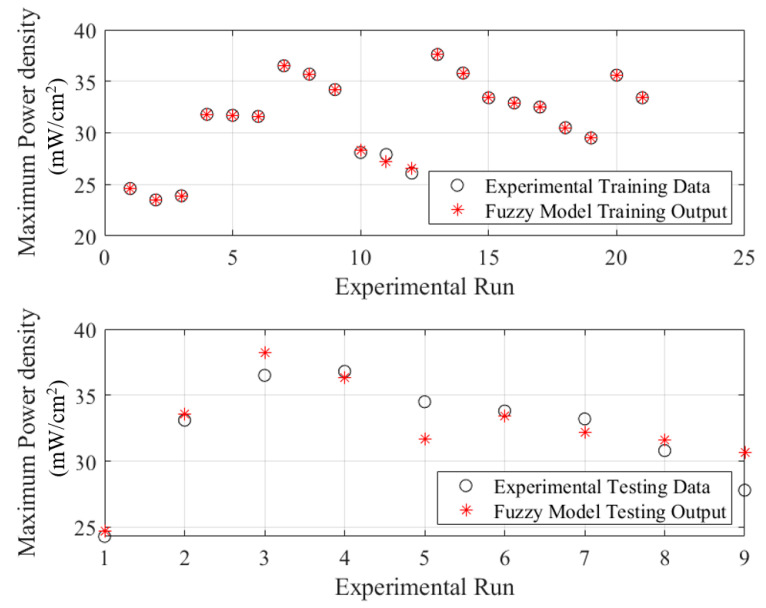
Experimental data versus predicted ones using fuzzy model of the DMFC.

**Figure 6:**
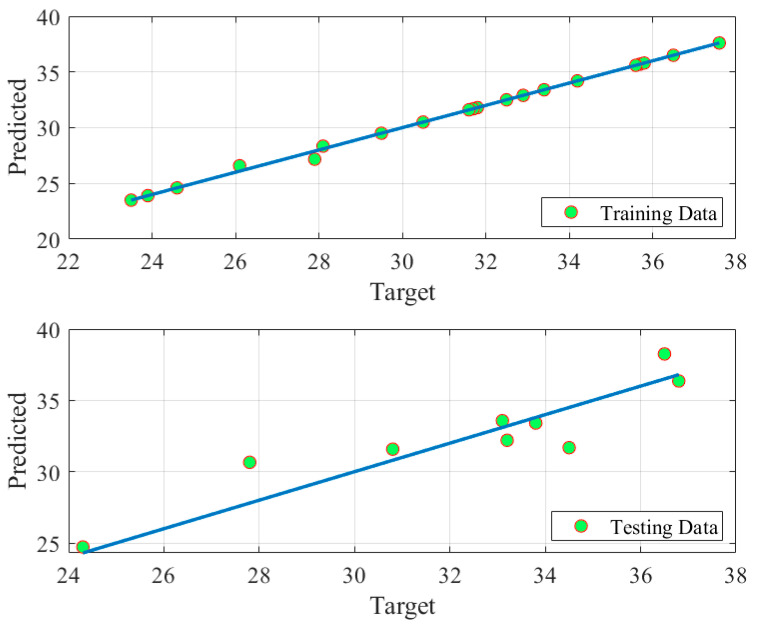
Prediction accuracy of fuzzy model of DMFC.

The developed fuzzy model can accurately estimate the power density of DMFC by detecting the accurate relationship between the controlling inputs and desired outputs of DMFC as seen in Figure 5. As clearly seen in the figure, a well matching between the estimated and experimental data. Additionally, an accuracy around 100% line was obtained for the prediction plots of the training and tested data obtained by the fuzzy model as demonstrated in Figure 6.

The next step after constructing the fuzzy model is the validation stage. Table 3 displays the validation results regarding the fuzzy model. Table 3 displays validation results of 70 °C, 1 M, and 300 mL/min for temperature, methanol concentration, and oxygen flow rate, respectively. Under these conditions, the maximum power density (MPD) values are 36.8 mW/cm^2^, 38.1 mW/cm^2^, and 36.35 mW/cm^2^ for experimental, RSM, and fuzzy modeling, respectively. As shown in Table 3, the percentage error decreased from 3.5% (ANOVA) to 1.22 using fuzzy modeling. 

After the validation stage, the fuzzy model was integrated with the BAS algorithm to detect the best operating conditions of the DMFC to maximize the power density. The optimization problem can be expressed in the following manner:

$$x=\underset{x\in R}{\arg}max(y)$$

*x* is the set of the input parameters, and *y* is power density of the DMFC.

**Figure 7:**
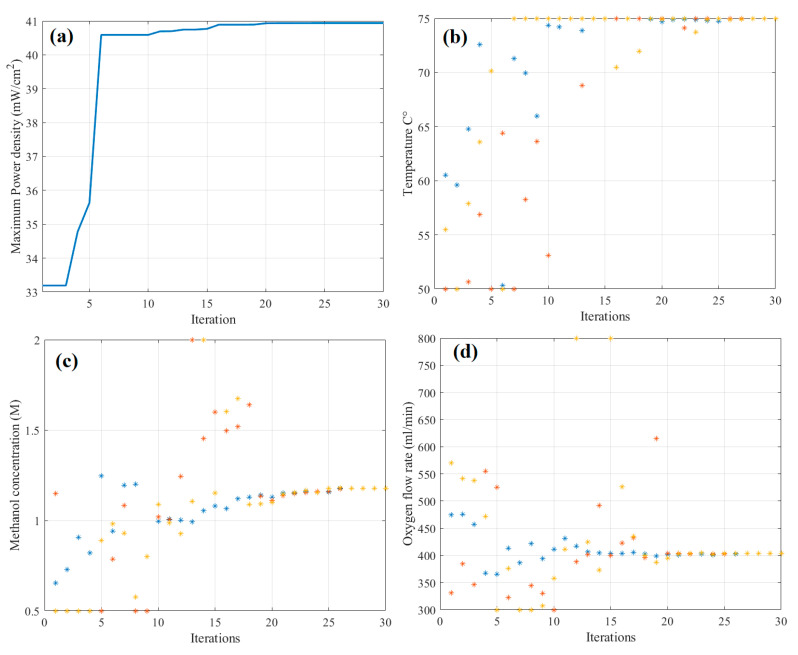
Particle convergence during the optimization: (**a**) best objective function, (**b**) temperature, (**c**) concentration of methanol, and (**d**) oxygen flow rate.

In Table 4, the optimal input parameters, and their corresponding power densities for the DMFC are presented using measured data, the RSM, and the BAS Algorithm. The combination of fuzzy modeling and the BAS algorithm resulted in an 8.88% and 7.5% increase in the power density of the DMFC when compared to the utilization of measured data and the RSM method, respectively. Figure 7 shows the particle convergence during optimization. As presented in Figure 7a, the best objective value of 40.94 (mW/cm^2^) was obtained at iteration no. 20. The optimal values for temperature, methanol concentration, and oxygen flow rate are 75 °C, 1.2 M, and 400 mL/min, respectively. The increase in performance with the increase in the methanol concentration from (1 M to 1.2 M) is related to the increase in the available methanol molecules at the anode surface for the electrochemical reaction and, especially, the increase in cell temperature from 70 to 75 °C. The increased methanol concentration increases the energy density, which is favorable for the commercial application of DMFCs. Improved performance was obtained at the same methanol flow rate, indicating no more flowing power requirements and at slight increase in the cell temperature. 

**Figure 8:**
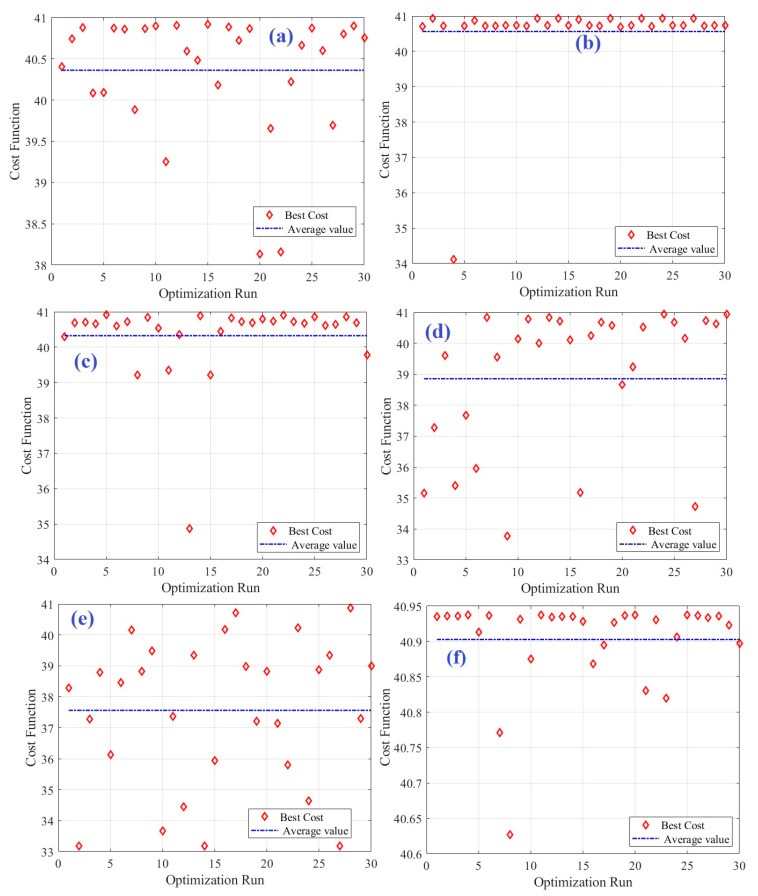
Details of the 30 runs: (**a**) SCA, (**b**) PSO, (**c**) JS, (**d**) HHO, (**e**) GA, and (**f**) BAS.

To confirm the power of the BAS algorithm, a comparison with SCA, PSO, GA, JS, and HHO was done. To avoid arbitrary results, all optimizers were executed 30 times, and comprehensive statistical evaluations were carried out. Table 5 and Figure 8 present the details of the 30 runs using the different optimizers. Various statistical metrics, including the best value, worst value, average value, and standard deviation, were computed and are presented in Table 6. The average cost function values (power density of DMFC) ranged between 40.9 and 37.56. BAS shows the highest average value of 40.9, followed by 40.56 in case of PSO, whereas GA showed the lowest value of 37.56. The STD values ranged between 0.07 and 2.38. BAS algorithm showed the best STD value of 0.07, followed by SCA (0.74), whereas as the lowest average value (2.38) was in case of using HHO. This demonstrates the superior performance of the BAS algorithm in identifying the best values associated with the maximum power output.

**Figure 9:**
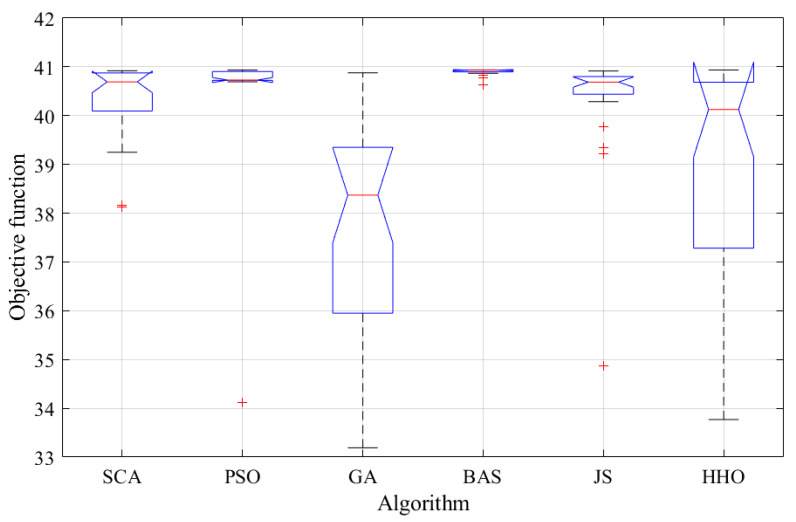
ANOVA ranking.

Other tests, such as an ANOVA and Tukey’s test, were also carried out. Table 7 outlines the results derived from using an ANOVA, while Figure 9 provides a visual representation of the rankings, effectively confirming the exceptional proficiency of the BAS method. The diagram highlights its superior average fitness and remarkable variances, reinforcing its position as the leading performer.

**Figure 10:**
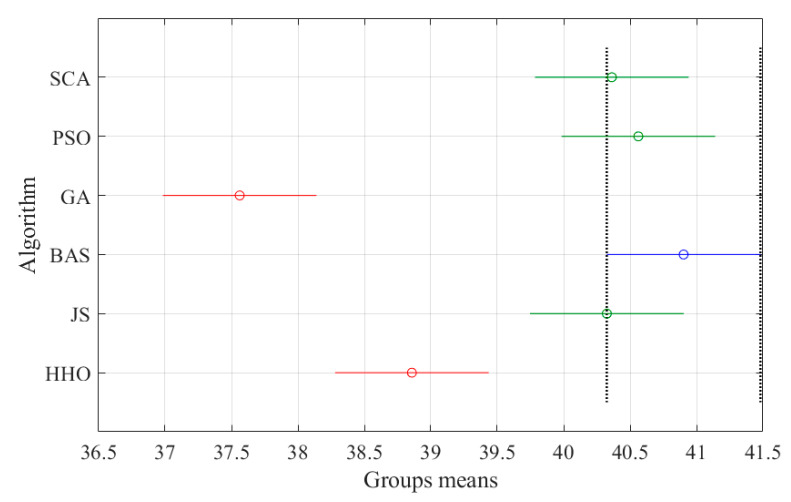
Results derived from using Tukey’s test.

The outcomes obtained through using Tukey’s test, depicted in Figure 10, validate the conclusions drawn from the ANOVA. Specifically, the mean results achieved by GA and HHO were distinctly inferior to those derived from using the BAS method. SCA, PSO, and JS showed the second highest performances, trailing behind the BAS method.

## 5. Conclusions 

The target of this work was to enhance the power density of a DMFC. A fuzzy model of a DMFC was developed based on collected data for temperature, methanol concentration, and oxygen flow rate. The best operating parameters to increase the power density of the DMFC were then found using the beetle antennae search (BAS) method. The RMSE values for the DMFC fuzzy model were 0.1982 and 1.5460 for the training and testing data, respectively. For the training and testing phases, the coefficient of determination (R^2^) values were 0.9977 and 0.89, respectively. The RMSE was reduced by 88% via the use of the BAS method compared to an ANOVA. It decreased from 7.29 using ANOVA to 0.8628 using fuzzy. The fuzzy model’s low RMSE and R^2^ values show that the modeling phase was successful. In comparison to the measured data and RSM, the combination of fuzzy modeling and the BAS algorithm increased the power density of the DMFC by 8.88% and 7.5%, respectively. To confirm the power of the BAS method, a comparison with SCA, PSO, GA, JS, and HHO was carried out.

The average values of the cost function (power density of the DMFC) ranged between 40.9 and 37.56. The BAS algorithm achieved the highest average value of 40.9, followed by PSO (40.56), while GA yielded the lowest average value of 37.56. Standard Deviation (STD) values varied between 0.07 and 2.38. The BAS algorithm exhibited the best STD value of 0.07, followed by SCA (0.74), with HHO recording the highest value of 2.38. This underscores the effectiveness of the BAS algorithm in deciding the best values corresponding to the highest power output.

## Figures and Tables

**Table 1:** Dataset points (with permission No. 5607770016240 from [46]).

Oxygen Flow Rate (mL/min)	Methanol Concentration (M)	Temperature (°C)	Maximum Power Density (mW/cm^2^)
300	1	50	24.6
500	1	50	24.3
600	1	50	23.5
800	1	50	23.9
300	0.5	60	31.8
500	0.5	60	31.7
600	0.5	60	31.6
300	1	60	36.5
500	1	60	35.7
600	1	60	34.2
800	1	60	33.1
300	2	60	28.1
500	2	60	27.9
600	2	60	26.1
300	1	75	37.6
500	1	75	36.5
600	1	75	35.8
300	0.5	70	33.4
500	0.5	70	32.9
600	0.5	70	32.5
800	0.5	70	30.5
1000	0.5	70	29.5
300	1	70	36.8
500	1	70	35.6
600	1	70	34.5
800	1	70	33.4
300	2	70	33.8
500	2	70	33.2
600	2	70	30.8
800	2	70	27.8

**Table 2:** Statistical metrics of the fuzzy model of the DMFC.

RMSE			*R* ^2^		
Train	Test	All	Train	Test	All
0.1982	1.5460	0.8628	0.9977	0.89	0.96

**Table 3:** Validation of fuzzy model of DMFC.

Method	Temperature °C	Methanol Concentration	Oxygen Flow Rate (mL/min)	Maximum Power Density (mW/cm^2^)	Percentage Error (%)
Exp. [46]	70	1	300	36.8	0.0
RSM [46]	70	1	300	38.1	3.5
Fuzzy	70	1	300	36.35	1.22

**Table 4:** Achieved best parameters using considered approaches.

Method	Temperature °C	Methanol Concentration	Oxygen Flow Rate (mL/min)	Maximum Power Density (mW/cm^2^)
Exp. [46]	70	1	300	37.6
RSM [46]	70	1	300	38.1
Fuzzy and BAS	75	1.2	400	40.94

**Table 5:** Details of the 30 runs.

No.	SCA	PSO	GA	BAS	JS	HHO	No.	SCA	PSO	GA	BAS	JS	HHO
**1**	40.41	40.7	38.28	40.94	40.29	35.16	**16**	40.18	40.9	40.17	40.87	40.44	35.18
**2**	40.74	40.93	33.19	40.94	40.69	37.28	**17**	40.89	40.73	40.71	40.89	40.82	40.25
**3**	40.88	40.72	37.27	40.94	40.71	39.6	**18**	40.72	40.72	38.98	40.93	40.72	40.68
**4**	40.09	34.12	38.78	40.94	40.66	35.41	**19**	40.87	40.94	37.2	40.94	40.69	40.58
**5**	40.09	40.72	36.14	40.91	40.92	37.68	**20**	38.13	40.69	38.83	40.94	40.8	38.67
**6**	40.87	40.87	38.46	40.94	40.6	35.96	**21**	39.66	40.73	37.14	40.83	40.73	39.24
**7**	40.86	40.73	40.15	40.77	40.72	40.84	**22**	38.16	40.94	35.8	40.93	40.91	40.53
**8**	39.88	40.73	38.83	40.63	39.21	39.55	**23**	40.22	40.7	40.23	40.82	40.72	33.96
**9**	40.87	40.73	39.49	40.93	40.84	33.77	**24**	40.66	40.93	34.64	40.91	40.68	40.93
**10**	40.9	40.73	33.67	40.88	40.54	40.14	**25**	40.87	40.73	38.87	40.94	40.85	40.69
**11**	39.25	40.72	37.37	40.94	39.35	40.79	**26**	40.6	40.73	39.35	40.94	40.62	40.16
**12**	40.91	40.94	34.45	40.93	40.35	40	**27**	39.7	40.93	33.19	40.93	40.64	34.73
**13**	40.59	40.73	39.35	40.93	34.87	40.84	**28**	40.8	40.72	40.88	40.94	40.85	40.73
**14**	40.48	40.93	33.19	40.93	40.88	40.72	**29**	40.9	40.73	37.29	40.92	40.7	40.62
**15**	40.92	40.73	35.95	40.93	39.21	40.11	**30**	40.75	40.73	39	40.9	39.77	40.94

**Table 6:** Results derived from the statistical evaluation of the considered optimizers.

	SCA	PSO	GA	BAS	JS	HHO
Best	40.92	40.94	40.88	40.94	40.92	40.94
Worst	38.13	34.12	33.19	40.63	34.87	33.77
Mean	40.36	40.56	37.56	40.9	40.33	38.86
STD	0.74	1.2	2.33	0.07	1.12	2.38

**Table 7:** ANOVA results.

Source	df	SS	MS	F	Prob
**Columns**	5	248.365	49,673	20.11	8.364 × 10^−13^
**Error**	174	429.835	2.470		
**Total**	179	678.200			

## Data Availability

Data are contained within the article.
